# Single Functionalized pRNA/Gold Nanoparticle for Ultrasensitive MicroRNA Detection Using Electrochemical Surface‐Enhanced Raman Spectroscopy

**DOI:** 10.1002/advs.201902477

**Published:** 2019-12-18

**Authors:** Taek Lee, Mohsen Mohammadniaei, Hui Zhang, Jinho Yoon, Hye Kyu Choi, Sijin Guo, Peixuan Guo, Jeong‐Woo Choi

**Affiliations:** ^1^ College of Pharmacy College of Medicine/Department of Physiology and Cell Biology/Dorothy M. Davis Heart and Lung Research Institute Ohio State University Columbus OH 43210 USA; ^2^ Department of Chemical and Biomolecular Engineering Sogang University 35 Baekbeom‐ro, Mapo‐gu Seoul 121‐742 Republic of Korea; ^3^ Department of Chemical Engineering Kwangwoon University 20 Kwangwoon‐Ro, Nowon‐Gu Seoul 01897 Republic of Korea

**Keywords:** biosensors, electrochemical surface‐enhanced Raman spectroscopy (EC‐SERS), microRNAs, monofunctionalization, pRNA 3WJ

## Abstract

Controlling the selective one‐to‐one conjugation of RNA with nanoparticles is vital for future applications of RNA nanotechnology. Here, the monofunctionalization of a gold nanoparticle (AuNP) with a single copy of RNA is developed for ultrasensitive microRNA‐155 quantification using electrochemical surface‐enhanced Raman spectroscopy (EC‐SERS). A single AuNP is conjugated with one copy of the packaging RNA (pRNA) three‐way junction (RNA 3WJ). pRNA 3WJ containing one strand of the 3WJ is connected to a Sephadex G100 aptamer and a biotin group at each arm (SEPapt/3WJ/Bio) which is then immobilized to the Sephadex G100 resin. The resulting complex is connected to streptavidin‐coated AuNP (STV/AuNP). Next, the STV/AuNP–Bio/3WJa is purified and reassembled with another 3WJ to form a single‐labeled 3WJ/AuNP. Later, the monoconjugate is immobilized onto the AuNP‐electrodeposited indium tin oxide coated substrate for detecting microRNA‐155 based on EC‐SERS. Application of an optimum potential of +0.2 V results in extraordinary amplification (≈7 times) of methylene blue (reporter) SERS signal compared to the normal SERS signal. As a result, a highly sensitive detection of 60 × 10^−18^ m microRNA‐155 in 1 h in serum based on monoconjugated AuNP/RNA is achieved. Thus, the monofunctionalization of RNA onto nanoparticle can provide a new methodology for biosensor construction and diverse RNA nanotechnology development.

## Graphical abstract

A monofunctionalized gold nanoparticle (GNP)/RNA structure is fabricated using a packaging RNA three‐way junction motif of the bacteriophage phi29 DNA packaging motor. The structure is further engaged to form a rigid λ‐shaped bioprobe RNA architecture onto the GNP–electrodeposited indium tin oxide coated substrate for highly sensitive and specific detection of microRNAs down to 60 × 10^−18^ m by means of electrochemical surface‐enhanced Raman spectroscopy.

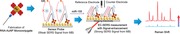

## 1 Introduction

RNA‐based nanotechnology provides the potential to generate millions of unique nanostructures that have therapeutic, biomedical, biosensing, and bioelectronic applications.^1^, ^2^ RNA molecules contain defined structures that serve as the building blocks of stem‐loops, which are motifs for intra‐ and intermolecular interactions of the dovetail joints used to fabricate the delicate nanostructure of these molecules.^3^ Moreover, aptamers, ribozymes, small interfering RNAs, microRNAs (miRNAs), and noncoding RNAs have unique functionalities that may be used to construct various nanostructure features.^4^ RNA nanotechnology is a rapidly growing field that involves the programmable and addressable design of 3D RNA structures.

**Figure 1:**
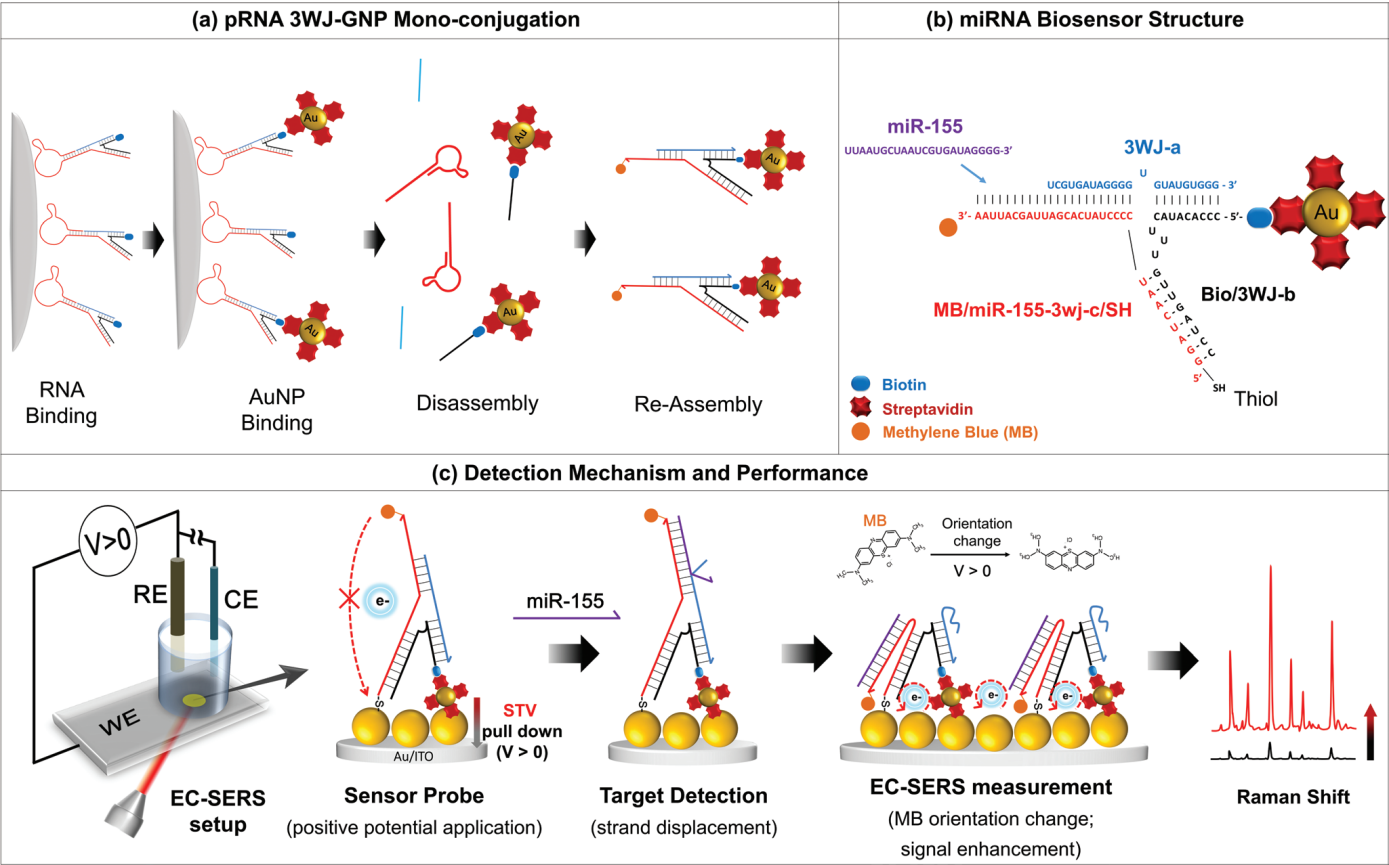
Schematic diagram showing the monofunctionalization of AuNP onto the RNA 3WJ structure alongside the biosensor structure and performance.

The programmable self‐assembly of simple building blocks such as nucleic acids, proteins, and nanoparticles that may be used to build highly ordered complex structures have recently received much attention in the field of nanotechnology.^5^ However, the programmability of nanoparticles such as gold, silver, and quantum dots that consist of crystals of atoms is challenging because of their almost spherical shape, large surface area, and anisotropic nature. This programmability requires the controllable functionalization of small molecules or macromolecules at specific locations on the surface of these nanoparticles with precise stoichiometric control. Efforts have been made to break the interaction symmetry between the nanoparticles and the surface particles and to achieve stoichiometry control by the selective functionalization of the biomolecules on the surface. Approaches based solely on stoichiometry require dilution conditions, resulting in a limited throughput. Some groups reported strategies to control the number of biomolecules on the surface of GNP to maximize its programmability using a ligand replacement approach.^6^ These strategies have broaden the potential applications of functionalized DNA nanoparticles in nanostructure construction, drug delivery, single‐molecule studies, and bioassay design. However, the selective functionalization of RNA is still required for investigation. Although, our group's previous study also showed the selective functionalization between RNA and quantum dot for memory device application,^7^ still, the monoconjugation method between RNA molecules and nanoparticles has not yet been reported. To investigate monoconjugation between RNA and nanoparticles, the present study introduced a packaging RNA (pRNA) three‐way junction (3WJ) motif, which was tagged with a sephadex G‐100 resin recognizing RNA aptamer and biotin (SEPapt/3WJ/Bio),^7^ to the Sephadex G100 resin. Streptavidin‐coated Au nanoparticles (STV/AuNPs) were bound to the SEPapt/3WJ/Bio through the streptavidin–biotin interaction (**Figure** **1**a). The STV/AuNP–SEPapt/3WJ/Bio conjugates were then dissociated with an elution buffer into three individual fragments: STV/AuNP–Bio/3WJb, 3WJa, and SEPapt/3WJc. The STV/AuNP–Bio/3WJb fragment was collected and purified. To quantify the binding ratio between STV/AuNP and pRNA 3WJb, the STV/AuNP–Bio/3WJb fragment was reassembled with Cy3‐labeled pRNA 3WJa and pRNA 3WJc fragments. The binding ratio was determined by a single‐molecule photobleaching (SMPB) assay. Moreover, to extend the RNA nanotechnology using this RNA–AuNP monoconjugate, we developed an ultrasensitive electrochemical surface‐enhanced Raman spectroscopy (EC‐SERS) biosensor to detect miRNA‐155 (miR‐155), which is overexpressed in MDA‐MB‐231 breast cancer cells and underexpressed in A549 lung cancer cells.^8^, ^9^ miRNAs as short endogenous noncoding generations of RNAs that exhibit abnormal expression in diverse organelles, have become important clinical biomarkers for the detection of various human cancers and diseases.^10^

To date, various miRNA detection methods have been proposed.^11^ For example, quantitative real‐time polymerase chain reaction (qRT‐PCR) is a well‐known standard method for detecting different target strands; however, limitations exist when attempting to detect shorter targets, such as miRNAs.^12^ Because of the shortness of miRNA sequences, the complicated primer design that is required to control their low melting temperature during amplification can cause low primer hybridization efficiency and cross contamination.^13^ To overcome those challenges, several signal‐amplifying approaches have been developed such as bio‐barcode assay,^9^ duplex specific nuclease,^14^ rolling circle amplification,^15^ and isothermal amplification.^16^ Despite their high sensitivity toward single base pair mutation, their labor intensiveness and multistep detection process have made the other methods more applicable approaches for the short RNA detection.

SERS technique as a very sensitive method has been widely used toward the accurate detection of miRNAs.^17^ For example, dual circular strand‐displacement polymerization SERS method,^18^ plasmonic nanowire interstice sensor,^19^ GNP hybridization,^20^ and inverse molecular sentinel nanoprobes^21^ have represented sound performances toward miRNA detection. Nevertheless, SERS measurement in the liquid dispersion is restricted to poor spectral reproducibility of SERS‐active nanoparticles, due to the agglomeration effect and difficulty in laser focusing. Therefore, SERS‐active nanopattern solid electrodes were introduced to resolve that challenge. To date, many solid electrode–based platforms have been developed and coupled with amplification methods including dual enzymatic amplification system,^22^ catalytic hairpin assembly amplification,^23^ and hollow Au/Ag integrated target‐catalyzed hairpin assembly.^24^ Although, despite their considerable contribution in improving the sensitivity and reproducibility of SERS‐based biosensors, they have limitations such as multiple and time‐consuming detection steps and more essentially, disordered spatial arrangement of the sensor probe, which is a very challenging issue in SERS‐based biosensors to achieve a reliable, fast, and ultrasensitive platform.

On the other hand, electrochemical biosensors as the fast, precise, and cost‐effective methods,^25^ have been considering as more efficient platforms for miRNA detection at ultralow concentration alongside the mismatch detection capability.^26^ However, instability and low sensitivity at low level of target concentrations because of nonlocalized and nonintense electrochemical signals need complicated detection process which cause cross contaminations. Notably, despite the sensitivity (low detection limit), specificity (mismatch discrimination), and simplicity of a biosensor, stability and reproducibility are more vital features to be seriously considered. Recently, we used RNA 3WJ to electrochemically detect miRNA with high sensitivity, nevertheless, the detection method required many steps and difficulties in sample preparation.^27^ In another study, we developed a platform to overcome the limitations of both electrochemical and SERS‐based miRNA detection methods by combining both the techniques from which SERS and EC were both measured independently.^28^ The platform however was not sensitive enough and represented a low reproducibility because of an uncontrolled immobilization process and nonaligned signal recording. Detailed analyses of the previously reported biosensors are provided in Table S1 (Supporting Information).

Here, to overcome those issues, a newly developed technology for one‐to‐one conjugation of AuNP and RNA 3WJ (AuNP/3WJ) was reported and applied for quantification of miRNA using an aligned EC‐SERS method that could detect 60 × 10^−18^ m of miRNA in 1 h with the ability to successfully discriminate between single base pair mutations. The rigid λ‐shaped RNA 3WJ structure was designed as the sensor probe and allowed sufficient spacing for target invasion and facilitating the electron transfer required for the EC‐SERS measurement. To construct the biosensor electrode, AuNPs were electrodeposited onto indium tin oxide coated (ITO) substrate (AuNP/ITO) (Figures S1 and S2, Supporting Information) and prepared for EC‐SERS experiments. To apply the monofunctionalized AuNP/3WJ to the miRNA biosensor, the 3WJb‐arm was modified to create a multifunctional group. 3WJc was prepared to detect miRNA, having a methylene blue (MB) group and a thiol (SH) group at opposing ends of the strand (MB/miR‐155‐3WJc/SH). The MB group is covalently bonded to the head of the probe and functions as both redox and Raman signal reporter. The SH group enabled the anchoring of the monofunctionalized AuNP/3WJ to the AuNP‐electrodeposited ITO substrate. The 3WJa was used as a displacer when the target miR‐155 was added (Figure 1b). As such, the proposed AuNP/RNA 3WJ can embody the five functions into the bioprobe.

The structure transformation resulting from the target hybridization and strand displacement, followed by the collision of MB onto the AuNP/ITO which was also in close vicinity to the monoconjugate AuNP, boosted the EC‐SERS signal of MB. We recorded the SERS signal emerging from MB while applying a constant optimal potential of +0.2 V to the electrode (Figure 1c). There were mainly three factors integrated in the high pace and ultrasensitivity of the proposed biosensor: i) SERS signal amplification of MB by ≈7 times due to the application of a constant potential of +0.2 V, ii) formation of a rigid λ‐shaped RNA chip (resulting from the applied positive potential, high stability of RNA/RNA hybrids compared with DNA/RNA,^2^ the presence of a loop in the junction and the high flexibility of the RNA compared with DNA strands), could cooperatively accelerate the structural switch, and finally iii) the monoconjugated AuNP, compared with the multifunctionalized AuNP, remarkably reduced the hindrance effects and provided an anchoring site for efficient collision of MB to the surface. Moreover, we could clarify the rationale behind the signal enhancement which was mainly because of the tethered AuNPs and the orientation of MB molecule.

## 2 Results and Discussion

### 2.1 Working Principle of the Biosensor

The prepared STV/AuNP–Bio/3WJb monoconjugate was used for the single‐step detection of miR‐155 based on the target strand‐displacement mechanism by means of the EC‐SERS technique. As shown in the schematic diagram in Figure 1b, the SH group– and MB group–modified miR‐155/3WJc (SH/miR‐155‐3WJc/MB) was immobilized onto the AuNP/ITO glass (optimization experiment is provided in Section S5 and Figure S1 in the Supporting Information). The STV/AuNP–Bio/3WJb monoconjugate was then hybridized to form the structure, prior to the hybridization of the 3WJa strand to produce the biosensor probe. We applied a constant potential of +0.2 V (optimal) to the AuNP/ITO electrode i) to pull down the STV/AuNP–Bio (at pH 7.4, streptavidin molecules with the isoelectric point of ≈5 represent a negatively charged STV/AuNP–Bio structure) and ii) to enhance the SERS signal of MB (will be discussed later in Section 2.5).

As a result, the AuNPs in the STV/AuNP–Bio/3WJb monoconjugate acted as an anchor for the probe structure for maintaining a firm and upright λ shape and for the SERS signal enhancement of MB. The laser was focused on the top side of the AuNP/ITO glass, and the resulting SERS signal was monitored and recorded in real time. Before the target hybridization, the tethered MB with flexible single‐stranded segment at the terminus is the farthest from the both surface and AuNPs or can be intercalated into the stiff RNA duplex form (due to the nature of MB), providing low EC or SERS signal. However, after the target hybridization, the flexible segment hybridizes with target sequence, and thus the intercalated MB is removed and collided to the surface due to the duplex structural conformation and abolishment of the 3WJ joint structure. This was nearest to the AuNPs and provided greater electron‐transfer facilitation and produced significant signals.

### 2.2 Conjugation and Characterization of AuNP–pRNA 3WJ Monoconjugate

**Figure 2:**
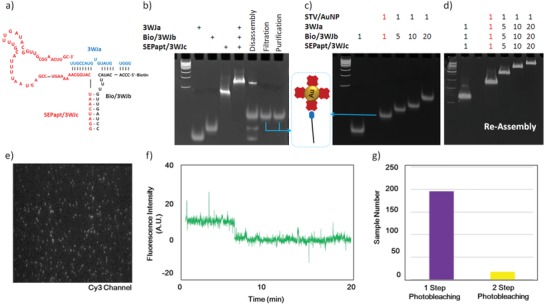
Monofunctionalization. a) 2D structure of the biotinylated RNA–3WJ containing an aptamer that can bind Sephadex G100; native PAGE gel (12% TBM) stained with ethidium bromide (EtBr) showing the assembly of b) SEPapt/3WJ/Bio (lane 5), the disassembly step after STV/AuNP hybridization (lane 6), filtration (lane 7), and purification (lane 8) of the STV/AuNP‐Bio/3WJb monoconjugate. c) STV/AuNP–Bio/3WJb monoconjugate (red letters) and multiconjugates with different ratios. d) Reassembly of STV/AuNP–Bio/3WJ monoconjugate (red letters) and multiconjugates with different ratios. Lane 1 is a 100 bp DNA ladder. e) Typical overlaid fluorescence image of the cy3‐labeled AuNP–3WJ monoconjugate. f) Photobleaching trace of the STV/AuNP–Bio/3WJ/Cy3 monoconjugate. g) The population distribution of the STV/AuNP–Bio/3WJ/Cy3 conjugate from the analysis of the number of photobleaching steps of the Cy3 fluorophore (data were extracted on the basis of the four independent experiments).

For the monoconjugation of AuNP and pRNA 3WJ, two functional modules, a Sephadex‐binding RNA aptamer and a biotin moiety, were integrated into one RNA nanoparticle using a pRNA–3WJ motif as a scaffold. **Figure** **2**a shows a schematic diagram of the designed SEPapt/3WJ/Bio pRNA nanostructure. Figure 2b shows the 12% native polyacrylamide gel electrophoresis (PAGE) confirming the formation of SEPapt/3WJ/Bio pRNA nanoparticles. The gel results show that the Sephadex aptamer and biotin moiety did not hamper the formation of 3WJ, as the pRNA–3WJ nanoparticle was assembled well with high affinity. Based on this, the STV/AuNP was conjugated to the SEPapt/3WJ/Bio. Lane 6 illustrates the disassembly step after the STV/AuNP hybridization before the filtration of the analytes. Clear bands can be seen in lanes 7 and 8, which represent the achievement of the STV/AuNP–Bio/3WJb monoconjugate. Moreover, we conducted a control experiment to ensure the monoconjugation of 3WJ and AuNP. The AuNP/Bio–3WJb monoconjugate was compared using different ratios of STV/AuNP and Bio/3WJb (1:5, 1:10, and 1:20). As illustrated in Figure 2c, increasing the ratio resulted in decreasing the migration speed of the macromolecule inside the gel. The obtained results further confirm the formation of the AuNP/Bio–3WJb monoconjugate. Moreover, the reassembly of the conjugated STV/AuNP–Bio/3WJb with 3WJa and 3WJc was confirmed by the 12% native tris‐borate magnesium (TBM) PAGE gel (Figure 2d). The STV/AuNP–Bio/3WJb monoconjugate was compared with a mixture of STV/AuNP and Bio/3WJ. Lane 4 shows the monofunctionalized STV/AuNP–Bio/3WJ. This migration shows the reassembly of 3WJ. Lanes 5–7 illustrate the mixtures of STV/AuNP and Bio/3WJ with different ratios (1:5, 1:10, and 1:20). The results show that the AuNP–3WJ monoconjugate (Figure 2d, lane 4) migrated faster than the mixture (Figure 2d, lanes 5–7). The results also indicate that the binding ratio between Bio/3WJb and STV/AuNP is less than 5:1. It is hypothesized that this originated from the steric hindrance between Bio/3WJ on G100 resin and STV/AuNP. Each STV has four binding sites for biotin.^29^ However, when the Bio/3WJb is immobilized on the G100 resin surface, the accessibility of the biotin moiety to STV/AuNP is restricted (Figure 1). The spherical nature of AuNP made it difficult to have multiple Bio/3WJb strands attached to the resin surface, thus making monoconjugation of STV/AuNP to Bio/3WJb achievable (refer to Figure S3 in the Supporting Information for morphology and size distribution of STV/AuNP). In contrast to the STV/AuNP–Bio/3WJb mixture, the Sephadex aptamer–based conjugation method provided site‐specific and stoichiometry‐defined conjugation, a unique consequence of conjugation.

### 2.3 Characterization of the AuNP–pRNA 3WJ Monoconjugate Using Single‐Molecule Fluorescence Microscopy

To verify monofunctionalization of the AuNPs with a pRNA 3WJ nanostructure more clearly, a SMPB assay was applied to the reassembled STV/AuNP–Bio/3WJ/Cy3 conjugate. The STV/AuNP–Bio/3WJ/Cy3 conjugate was immobilized in biotin‐coated glass coverslips for single‐molecule total internal reflection fluorescence imaging. Each STV/AuNP–Bio/3WJ conjugate appeared as an individual fluorescence spot in the image (Figure 2e). A photobleaching assay was applied to determine the copy number of RNA on the AuNPs conjugated within a single complex. As the single fluorophores possess quantized photobleaching characteristics, the number of fluorophores within a diffraction‐limited fluorescent spot can therefore be deduced from the number of decreasing steps of fluorescence intensity over time. On the basis of this principle, the number of Cy3 molecules within each STV/AuNP–3WJ conjugate was determined by counting the photobleaching steps (Figure 2f). Based on 4 times of experiments, a total of 213 fluorescent spots were analyzed, and a histogram of the photobleaching steps was used to summarize and display the population distribution (Figure 2g). About 92% of the fluorescent spots displayed a one‐step drop in their photobleaching traces, indicating the existence of only one Cy3 fluorophore and therefore one 3WJ construct within each individual conjugate. Only a small amount of the spots displayed two‐step photobleaching, which could be due to two conjugates residing in close proximity to each other within the diffraction limit. These results show that the RNA aptamer–based site‐specific conjugation method can produce highly homogeneous monoconjugates.

### 2.4 Structure Analysis of 3WJ before and after the Target Hybridization

**Figure 3:**
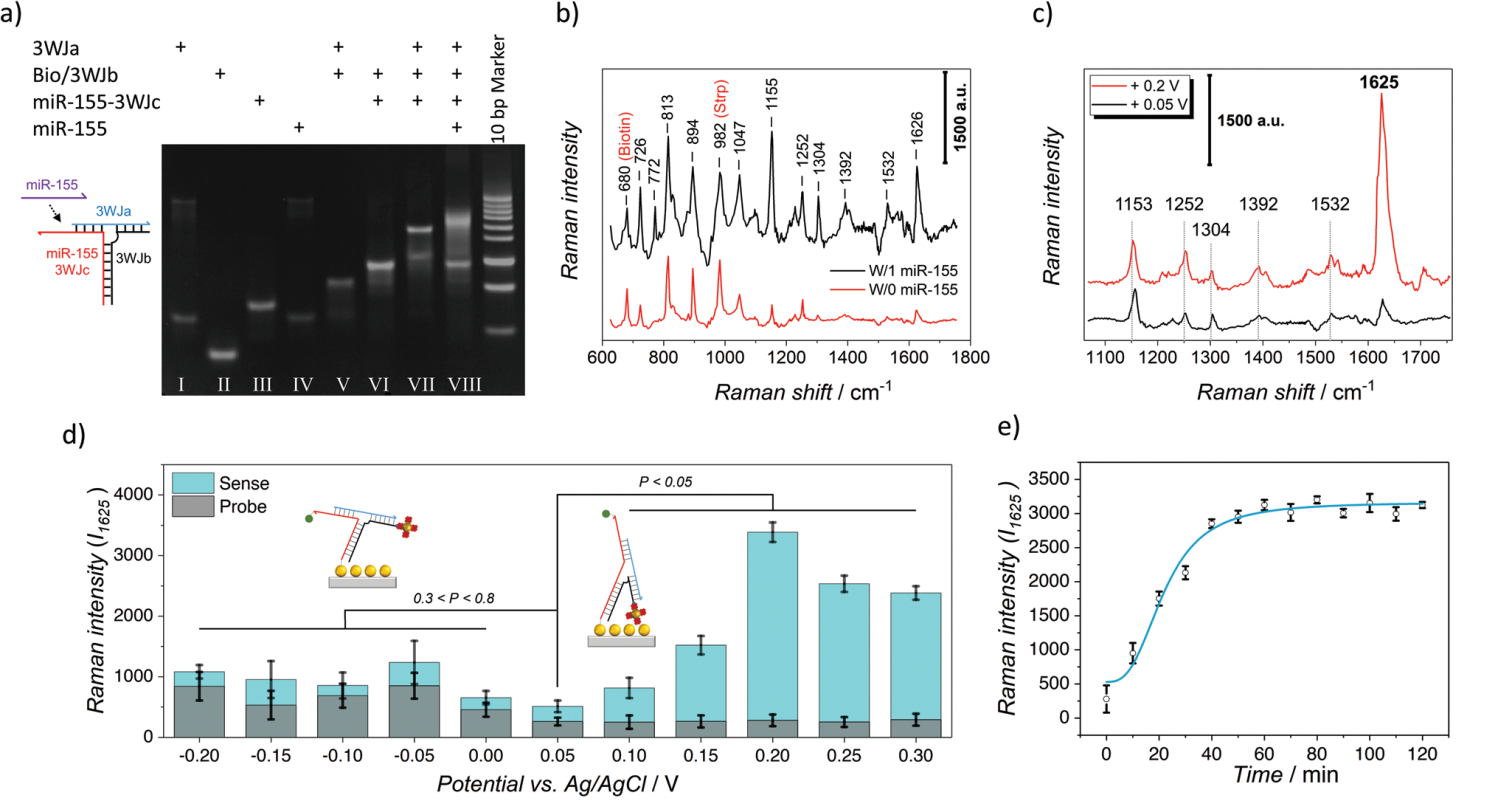
Sensing mechanism. a) Native PAGE gel stained with EtBr showing the biosensor structure transformation upon the target invasion. b) Representative EC‐SERS spectra of the 3WJ biosensor under the constant potentials of +0.05 V before (W/0) and after (W/1) the miR‐155 invasion. c) Comparison between EC‐SERS spectra of the adsorbed miR‐155 (100 × 10^−12^ m) on the sensor recorded under the constant potentials of +0.05 and +0.2 V. d) Statistical comparison between the Raman intensity at 1625 cm^−1^ for the adsorbed miR‐155 (100 × 10^−12^ m) as a function of the electrode potential. Data were averaged from ten samples. e) Raman intensity at 1625 cm^−1^ recorded under a constant potential of +0.2 V as a function of the incubation time of the target miR‐155 on the sensor. The signals were collected at 1 s exposure times using a 785 nm laser with the spot size of 1 µm^2^. Data were extracted on the basis of the average signal of 50 points of three independent samples.

To confirm the structural transformation feasibility of the developed 3WJ, TBM PAGE was used. As shown in **Figure** **3**a, lanes 1–4 illustrate the strands 3WJa, Bio/3WJb (without AuNP), unmodified miR‐155‐3WJc, and miR‐155, respectively. Lanes 5 and 6, respectively, correspond to 3WJa/3WJb and 3WJb/miR‐155‐3WJc hybridization, for which we observed two distinct and bright bands indicating good hybridization efficiency. The probe structure, which consisted of the three strands of 3WJa, Bio/3WJb, and miR‐155‐3WJc, was well‐formed and could be seen in lane 7. After hybridization of the target strand (miR‐155) with the sensor probe, a bright band (lane 8) was observed at higher levels compared with the probe (lane 7); this implies the occurrence of strand displacement and successful structure transformation.

### 2.5 Detection of miR‐155 Using EC‐SERS Method

At first, our hypothesis was to apply a constant positive potential on the electrode surface in order to achieve a rigid λ shape architect of 3WJ. We used +0.05 V as the applied potential and recorded the SERS signal before and after the target invasion. The target strand (miR‐155) was added to the 3WJ‐modified AuNP/ITO substrate and incubated for 1 h at room temperature. After cleaning, the SERS experiment was conducted in phosphate‐buffered saline (PBS) (pH 7.4) and the average signal from at least 50 random spots was calculated. Figure 3b illustrates typical SERS spectrums recorded from the developed biosensor in the absence (bottom/red) and the presence (top/black) of the target miRNA.

We observed a weak MB Raman signal in the absence of miR‐155, as the signals mostly arose from the RNA molecular structure and biotin–streptavidin molecules. While, in the presence of the target species, there was a pronounced increase in the intensity of the MB Raman bands, demonstrating the viability of our strategy (Figure 3b). Despite the presence of the fluorescence activity of MB in the samples and a number of invariant weak Raman bands from ≈400 to ≈1150 cm^−1^ that resulted from ribose and phosphate residues,^30^, ^31^, ^32^, ^33^, ^34^, ^35^, ^36^, ^37^, ^38^ high‐quality Raman spectra were recorded. However, the Raman bands at ≈680 and ≈962 cm^−1^, which are assigned to biotin and streptavidin,^39^ were almost invariant with the structure transformation demonstrating the rigid λ shape architect of 3WJ. The Raman bands observed at ≈772, 894, 1155, 1304, 1392, and 1626 cm^−1^ originated from MB^40^ and increased dramatically upon introducing the miR‐155, which further confirmed the structural transformation of the system. The Raman band recorded at ≈726 cm^−1^ is reported to be assigned to adenine residue,^31^, ^32^, ^37^, ^39^ and the one arising from ≈813 cm^−1^ is assigned to O—P—O stretching.^32^, ^33^, ^34^, ^36^, ^37^, ^39^ We observed another Raman band at ≈1047 cm^−1^, which has been previously assigned to P—O stretching and sugar phosphate —C—O— stretching.^31^, ^35^ The Raman band at 1252 cm^−1^ has been dedicated to adenine, cytosine, and uracil^31^, ^32^, ^36^, ^37^, ^38^ and is thought to originate from the adenine vibrations. Accordingly, the Raman band at 1532 cm^−1^ can be allocated to adenine, guanine, and cytosine stretching.^31^, ^34^, ^37^, ^39^

We also performed the electrochemical impedance spectroscopy (EIS) technique to verify the performance of the developed biosensor. The EIS experiment was carried out at the constant potential of +0.05 V in PBS (10 × 10^−3^ m, pH 7.4) containing 5 × 10^−3^ m [Fe(CN)_6_]^3−/4−^ and 0.1 m KCl. As it is evident in Figure S4 (Supporting Information), the *R*_ct_ of bioprobe (≈384.2 Ω) increased to ≈443.6 Ω after hybridization with target miRNA. This might result from two factors: i) switching the structure would lead to more surface coverage and further blockage of the redox couple to reach the surface, also ii) addition of miRNA would impose more negative charge to the surface to repel the redux couple and hamper their diffusion efficiency.

#### 2.5.1 Optimal Potential Assessment for EC‐SERS

We also examined different constant electrode potentials and recorded the corresponding SERS signals. Interestingly, we observed a dramatic increase in the magnitude of the Raman band at 1625 cm^−1^ upon the application of the potential of +0.2 V to the electrode, while the other bands remained almost unchanged (Figure 3c). The rationale behind the observed phenomena is explained later in this section. Figure 3d shows the variation of EC‐SERS signal intensity at 1625 cm^−1^ as a function of applied constant potential. Interestingly, increasing the electrode potential from +0.05 V to more positive values was followed by enhancement of SERS signal where it reached its maximum value at +0.2 V being about 7 times higher than that of the initial value. The signal further decreased by increasing the potential. Notably, at the positive potential ranges, the recorded signal for the probe structure (before the target hybridization) was remarkably constant (vs control/+0.05 V), illustrating the robust λ structure of 3WJ, while in the negative region, it was highly unstable and unreliable. It should be mentioned that, since the Raman signal of MB was not very distinguishable before 1100 cm^−1^ and in order to eliminate the effects of the biotin and streptavidin Raman bands, as well as those possibly arising from human serum, the Raman window was chosen to be 1150–1750 cm^−1^ and the rest of the experiments were performed in this range.

On the other hand, sweeping the potential to zero and more negative values resulted in random changes in the recorded signals. As evident, the signals recorded for probe and sense are slightly different with low degree of significance versus control. This might be due to the fact that, application of positive potential to the surface is followed by detachment of the STV/AuNP–Bio from the surface to station the structure in an unstable figure which undoubtedly provides false and nonreproducible signals.

In addition, comparing the recorded sense signals of the negative and positive potential regions, the overall signals in negative region is much lower than that of the positive region. This might result from the engagement of tethered AuNPs in the SERS signal enhancement by incorporation with the AuNP/ITO substrate (schematic diagram of Figure 1c). Presumably, a comprehensive experiment is required to assure the role of small‐sized tethered AuNPs (3 nm) in SERS signal enhancement.

To further confirm our hypothesis, we recorded cyclic voltammograms of the electrodes for ten cycles. As seen in Figure S5a (Supporting Information), random changes in redox peaks' intensity as well as the location of different voltammograms can be clearly observed, demonstrating the orientation change in the 3WJ biosensor structure under different potentials during the voltage swiping. To further explore the electrochemical behavior of the system, differential pulse voltammetry (DPV) was used under different concentrations of the target miR‐155. As it is shown in Figure S5b (Supporting Information), the biosensor could not display a perfect performance under linear increment of the analyte concentrations. Disordered DPV signals as well as random shifts in the cathodic potentials were observed. Moreover, the calibration curve in Figure S5b (Supporting Information) illustrates relatively large error bars together with insignificant linearity (*R* ^2^ = 0.908) of the biosensor toward serial concentrations of miR‐155. The achieved data can supplement our proposed explanation for the necessity of application of positive potential to the electrode to keep the structure rigid and λ‐shaped.

In fact, the potential dependence of the Raman signal is reported to be due to the changes in the electronic distribution of the adsorbed molecule, surface orientation of the adsorbate, and the enhancement of the double‐layer electric field.^41^ Comparison of the two curves recorded for +0.05 and +0.2 V in Figure 3c shows emergence of new Raman bands and others underwent small shifts (1155–1153 and 1626–1625 cm^−1^). This might be due to the charge density dependency of the plasmon frequency, according to the following equation^42^

(1) $$\omega_{p}=4\pi ne^{2}\text{/}m\varepsilon_{0}$$

where *m* and *e* are the mass and charge of an electron, *n* is the density number of the free electrons, and ε_0_ is the vacuum permittivity. It is evident that the variation of *n* results in a plasmon frequency shift, resulting in a change in the local optical electric field at the metal surface.

Also, Lombardi and co‐workers reported the dependence of Raman band intensities of *p*‐nitrobenzoic acid on the applied potential where the maximum SERS signal was observed within the redox potential region of the species, possibly because the laser stimulates the static electric field of the metal electrode at the Fermi level to make photoemitted electron transfer possible.^43^ However, in our case, the signal enhancement was observed far beyond the redox peaks of the MB (between −0.3 and −0.5 V). As a result, we speculated that the positive charge of MB might be an important factor to consider. Application of positive potential might alter the orientation of MB on the surface, such that at a certain potential (+0.2 V), the C—C stretching and C—N—C skeletal bending (known to have very strong Raman intensities around 1600–1650 cm^−1^)^40^, ^44^ located at the aromatic rings of the MB would be positioned in a specific spot within the structure and AuNPs to provide a substantial signal enhancement at 1625 cm^−1^. In another report by Lombardi and co‐workers,^45^ the potential dependence of pyridine's SERS spectra at two specific bands of 1034 and 1004 cm^−1^ was attributed to a possible biomolecular coupling reaction and formation of a new structure, although, the first hypothesis is closer to our system configuration.

#### 2.5.2 Optimal Assembly Time Assay

The Raman intensity of 1625 cm^−1^ (*I*_1625_) at the fixed potential of +0.2 V was chosen as the detection signal of the developed 3WJ biosensor. The optimal assembly time assay for the miR‐155 hybridization to the 3WJ biosensor was performed to examine the detection speed of our proposed sensor. The EC‐SERS spectra derived from the 3WJ biosensor were collected from 0 to 120 min after being introduced by miR‐155 at a concentration of 100 × 10^−12^ m. The EC‐SERS intensity at 1625 cm^−1^ was monitored and plotted as a function of the incubation time (Figure 3e). It was evident that during the first 40 min of the incubation, the Raman intensity and MB reduction current rapidly increased to almost 70% of their final signals, and then gradually increased to a relatively stable plateau at >60 min. This indicates that nearly all of the target miR‐155 has been hybridized to the sensor probe, which further confirms the function of the monoconjugated nanoparticles in facilitating the fast structure switch, resulting in decreasing the detection time of our proposed biosensor. Therefore, the optimum detection time was chosen to be 60 min.

#### 2.5.3 Detection of Single Base Pair Mutations

**Figure 4:**
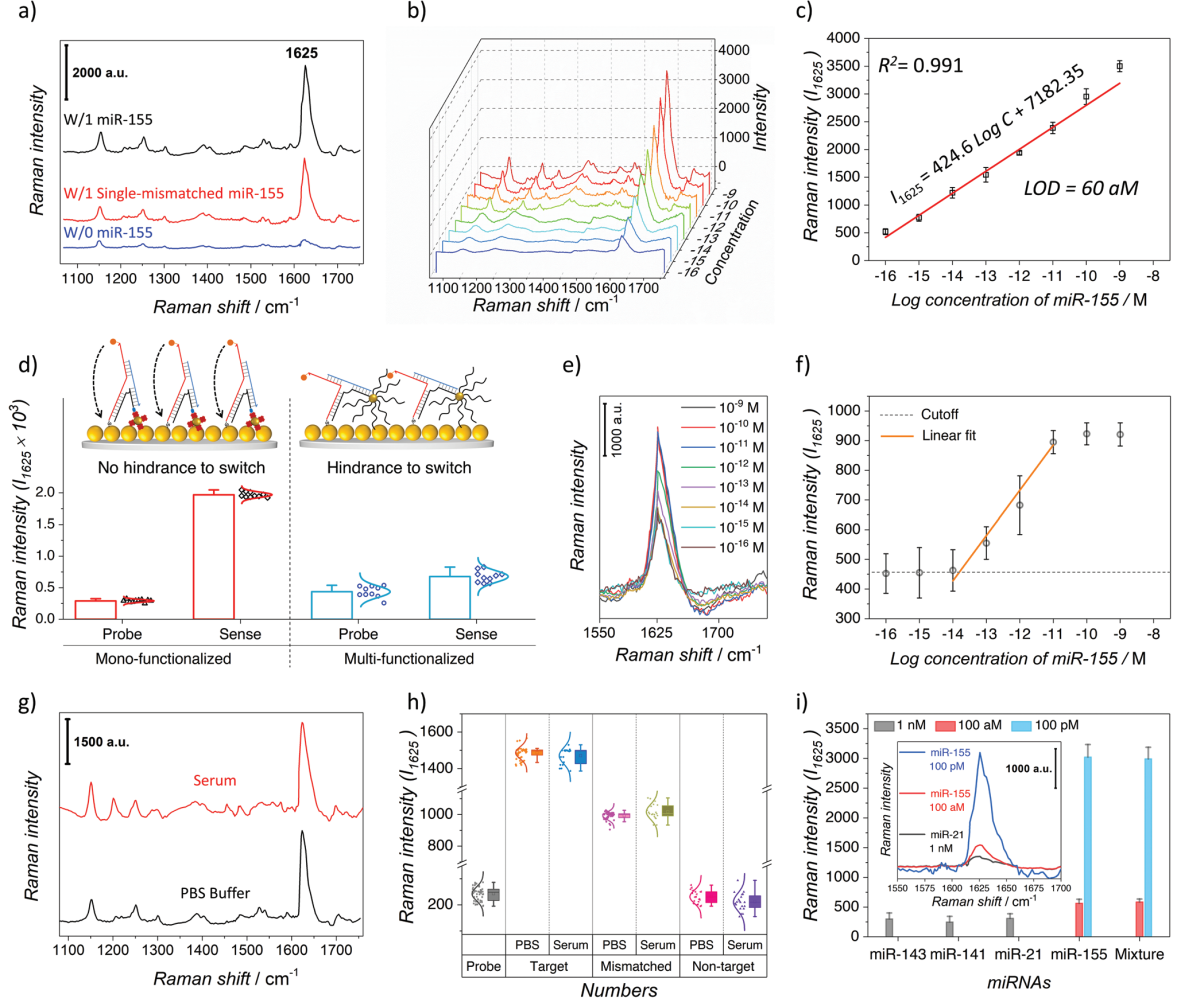
Sensing performance. a) Typical EC‐SERS spectra of 3WJ biosensor at +0.2 V before (blue) and after incubation with 100 × 10^−12^ m single‐mismatched (red) and 100 × 10^−12^ m complementary miR‐155 (black). b) EC‐SERS spectra obtained at concentrations of miR‐155 from 100 × 10^−18^ to 1 × 10^−9^ m. c) Linear correlation between the EC‐SERS signal intensity at 1625 cm^−1^ and the concentration of miR‐155. d) The corresponding statistical data recorded from the EC‐SERS signal at 1625 cm^−1^ for the sensors prepared by STV/AuNP–Bio/3WJb monoconjugate (left panel) and STV/AuNP–Bio/3WJb multiconjugate (right panel) after hybridization with 1 × 10^−12^ m target miR‐155; error bars and distribution curves obtained from ten different samples prepared under identical condition. e) EC‐SERS spectra obtained from multiconjugate biosensor at concentrations of miR‐155 from 100 × 10^−18^ to 1 × 10^−9^ m. f) The corresponding correlation curve for (e); error bars calculated based on three identical experiments. g) Representative EC‐SERS spectra of the 3WJ biosensor after being incubated with 100 × 10^−12^ m miR‐155 in a PBS buffer (black) and human serum (red). h) Statistical analysis and distribution curves of the EC‐SERS signal intensity at 1625 cm^−1^ of the 3WJ biosensor at +0.2 V before (Probe) and after hybridization with 100 × 10^−15^ m miR‐155 (Target), 100 × 10^−15^ m single‐base mismatched miR‐155 (Mismatched), and 1 × 10^−12^ m noncomplementary miRNA (Nontarget). Data were obtained from 50 random spots on the sensor chip for the PBS and serum samples, respectively. i) Variation in the EC‐SERS signal intensity at 1625 cm^−1^ upon introduction of different targets of 100 × 10^−12^ and 100 × 10^−18^ m perfectly matched miR‐155 and 1 × 10^−9^ m of nontarget miRNAs (miR‐21, miR‐141, and miR‐143); the inset illustrates typical SERS spectra from three corresponding targets. The signals were collected at 1 s exposure times using a 785 nm laser.

The specificity of the 3WJ biosensor toward a mismatch mutation was examined by introducing single‐base mismatched miR‐155 at 100 × 10^−12^ m to the sensor chip. As seen in **Figure** **4**a, in the absence of the target strand (W/0 miR‐155), a very weak Raman spectra was observed, while a pronounced increase in *I*_1625_ was seen after 1 h of incubation with perfectly matched miR‐155 (W/1 miR‐155). In the case of single‐base mismatched miR‐155, the change in *I*_1625_ was clearly lower than that of the complementary miR‐155 (≈31% decay). The reason for this decreased Raman signal is that i) the mismatched miRNA cannot effectively perform the strand displacement that follows the structure transformation; also ii) single mismatched duplexes have higher bending elasticity than the fully matched strands followed by an ineffective attachment of MB to the plasmonic AuNP/ITO surface. The obtained results indicate that the 3WJ biosensor represents a sound specificity toward discriminating the single‐mismatched and perfectly matched target strands.

In addition, in order to test the uniformity of the 3WJ biosensor, EC‐SERS spectra were recorded from 50 different hot spots on the sensor platform in both the absence and presence of 100 × 10^−15^ m target miR‐155, as well as single‐base mismatched miR‐155. The corresponding spectra are provided in Figure S6 (Supporting Information). Also in Figure S7 (Supporting Information), two Raman maps of the sensor chip show the EC‐SERS hot spots of the sensor probe before (Figure S7a, Supporting Information) and after 1 h of incubation with 10 × 10^−15^ m miR‐155 (Figure S7b, Supporting Information). They display very uniform hot spots, which indicate that almost all of the λ‐shaped probe structures have been switched. These results demonstrate a remarkable uniformity of the 3WJ biosensor.

#### 2.5.4 Limit of Detection

The dynamic detection range of the as‐prepared biosensor was also evaluated. As is clearly seen in Figure 4b and the corresponding Figure 4c, a wide dynamic range and linear correlation between the *I*_1625_ and the miR‐155 concentration from 100 × 10^−18^ to 1 × 10^−9^ m was achieved with the regression equation of *I*_1625_ = 424.6log*C* + 7182.35; *R* ^2^ = 0.991. The limit of detection (LOD) of the 3WJ biosensor was calculated to be 60 × 10^−18^ m, which is 3 times larger than the standard deviation of the background signal of the probe EC‐SERS spectra (*S*/*N* = 3). We also compared our obtained data with quantitative qRT‐PCR results (Supporting Information). As seen in Figure S8 (Supporting Information), a similar detection performance was observed. However, for the qRT‐PCR, the LOD for the miR‐155 detection was measured as 2.5 × 10^−15^ m. In addition, decreasing the miR‐155 concentration from 10 × 10^−15^ m resulted in deviation in the linear detection range.

#### 2.5.5 Role of AuNP–RNA 3WJ Monoconjugate

Although, we have developed a RNA–AuNP monoconjugate which would receive intensive interests from wide range of research disciplines, as a proof of concept, we employed this interesting structure for miRNA detection. In our research, we used STV/AuNP–Bio/3WJb monoconjugate to mainly resolve the hindrance effects faced by electrode modifications for reliable and reproducible detection of nucleic acids. Moreover, despite the small size of the tethered AuNPs, they represented a considerable impact on the SERS signal arising from MB.

In order to illustrate the importance of RNA–AuNP monoconjugate on our biosensor performance, we conducted a control experiment by comparing the STV/AuNP–Bio/3WJb monoconjugate and uncontrolled STV/AuNP–Bio/3WJb multiconjugate incorporated in the 3WJ motif. As seen in Figure 4d, after the target hybridization, the sensor prepared with STV/AuNP–Bio/3WJb monoconjugate displayed a higher SERS signal with a low relative standard deviation (RSD) of 4.7%, whereas, the sensor prepared with uncontrolled multiconjugate, STV/AuNP‐Bio/3WJb represented a lower signal with a high and unreliable RSD of 17.3%. This might be resulted from the hindrance effect of multiple RNA segments on the AuNP to hamper the structure transformation of 3WJ. Moreover, in the absence of the target, the probe signal of multiconjugate sensor was considerably higher than that of the monoconjugate sensor because of the demolished λ architect which further demonstrates the importance of STV/AuNP–Bio/3WJb monoconjugate in our sensing mechanism.

Moreover, we verified the multiconjugate biosensor by analyzing its performance toward serial dilutions of miR‐155. 24 electrodes consisting of eight groups of three sample sets were prepared using STV/AuNP–Bio/3WJb multiconjugate to be engaged for the detection of various concentrations of miR‐155. As it is illustrated in Figure 4e,f, compared to the monoconjugate biosensor (Figure 4c), the multiconjugate biosensor could work in a considerably shorter range of target concentration (10 × 10^−15^–10 × 10^−12^ m), from which the signal intensity (*I*_1625_) increased by only two orders of magnitude. This might be due to the fact that the monofunctionalized AuNP provides enough space for structural transformation, while the conventional one with a vast number of RNA strands hinders the structure to be switched upon introducing the target miRNA. In addition, the low sensitivity of multiconjugate biosensor would be because of high signal of the sensor probe before the target invasion, which resulted from the lower distance of MB from the electrode. Furthermore, due to the special arrangement of the multiconjugate probe on the surface, there would be less probe structures on the electrode, creating an early signal saturation at 10 × 10^−12^ m of miR‐155 (schematic diagram of Figure 4d).

#### 2.5.6 Sensing Performance in Serum

To further test the miRNA detection ability of the 3WJ biosensor in the human serum, miR‐155 was diluted in the 5% and 10% human serum and analyzed. Figure 4g illustrates a typical EC‐SERS spectrum from the 3WJ biosensor after being introduced to the 100 × 10^−12^ m miR‐155 in PBS buffer at pH 7.4 (black) and 5% human serum (red). The spectra are almost identical except for the appearance of a band at 1201 cm^−1^, which may result from the serum matrix; however, the EC‐SERS intensity at 1625 cm^−1^ remained relatively unchanged, indicating the good capability of our sensor in detecting miRNA in real samples. A small shift in the recorded signal at 1625 cm^−1^ would be due to the presence of different biomolecules in the serum.^8^, ^38^ A statistical experiment was performed to compare the response of the 3WJ biosensor toward target miR‐155 (100 × 10^−15^ m), single‐mismatched miR‐155 (100 × 10^−15^ m), and nontarget (miR‐21 at 1 × 10^−12^ m) in the PBS buffer and serum. The results are depicted in Figure 4h, wherein similar results between the PBS and serum samples, as well as distinctive EC‐SERS signals at 1625 cm^−1^, are observed. In addition, we investigated the potency of the 3WJ biosensor for the recovery of different concentrations of miRNA in the human serum, using the standard addition method. The data obtained from three different samples are tabulated in Table S2 (Supporting Information), from which a recovery of 97.2–106.0% for 5% serum and 96.9–104.1% for 10% serum were observed. This further demonstrates an acceptable performance of the 3WJ biosensor in miRNA detection in human serum.

#### 2.5.7 Reproducibility, Selectivity, and Stability Experiments

Reproducibility test was investigated by recording the EC‐SERS spectra at +0.2 V from five different sensors prepared under identical conditions and immobilized by 100 × 10^−12^ m miR‐155 for 1 h. The EC‐SERS spectra were collected and averaged from 50 random spots for each sample (Figure S9a, Supporting Information). The corresponding *I*_1625_ is shown in Figure S9b (Supporting Information), which demonstrates a small deviation of 6.9% and proves a sound reproducibility of the proposed biosensor. Additionally, a selectivity test was conducted to detect miR‐155 in the presence of different interfaces. The EC‐SERS spectra were collected after incubation of biosensor with miR‐21, miR‐141, and miR‐143 at 1 × 10^−9^ m and miR‐155 at 100 × 10^−12^ and 100 × 10^−18^ m, as well as a mixture of all four miRNAs. As clearly shown in Figure 4i, *I*_1625_ of MB for all of the interfaces were around the probe signal, and the signals obtained from the mixture solution was similar to that of miR‐155 alone. Additionally, a reusability experiment was carried out after a storage period of 1 month at 4 °C in a humid chamber, of which the sensor retained 97.8% and 95.7% of its initial electrochemical and EC‐SERS responses, respectively, showing the good reusability of our proposed biosensor. Moreover, for the stability test, five different sensors were prepared under identical experimental conditions and directed to the EC‐SERS measurement. After recording the EC‐SERS, the electrodes were rinsed with distilled water and dried under N_2_ gas before keeping in a 4 °C humid and dark chamber. Every other days, the samples were engaged to the EC‐SERS analysis and stored, as mentioned above. As shown in Figure S9c (Supporting Information), after 8 days, the samples still represented reliable signals being around 97.4% of their initial values to demonstrate the sound stability of our 3WJ biosensor.

## 3 Conclusion

A novel methodology for monofunctionalization of AuNPs with the single copy of RNA was developed possessing various therapeutic, biomedical, biosensing, and bioelectronic applications. As a proof of concept, the structure was applied for miRNA detection using the EC‐SERS technique. Each AuNP holds single copy of the pRNA 3WJ motif via the streptavidin–biotin reaction. The monofunctionalization of AuNP onto pRNA 3WJb was achieved by utilizing the RNA aptamer binding to sephadex G100 resin. This AuNP/3WJ fragment was then reassembled to evaluate the miRNA biosensor performance. An ultrasensitive biosensor with a single mismatch detection of miRNA‐155 achieved in only 1 h in human serum was constructed. The detection mechanism was based on the strand displacement strategy following the structural transformation, which brought MB into the vicinity of the AuNPs and the substrate, resulting in a remarkable increase in SERS signals. The monofunctionalized AuNP–RNA sustained a robust and λ‐shaped structure, removed the hindrance effects, and provided enough space for a prompt and accurate structural switch after introducing the target analyte. By means of the EC‐SERS approach, the SERS signal obtained from MB was shown to be reliant on the substrate potential, which was boosted at 1625 cm^−1^ by a magnitude of about sevenfold upon the application of +0.2 V. The principle behind the mechanism was revealed to open a new horizon for more interesting researches to come. A very low limit of detection of 60 × 10^−18^ m was achieved with sound linear detection range from 100 × 10^−18^ to 1 × 10^−9^ m and a vigorous ability to detect single mutations. The proposed AuNP/RNA monoconjugate biosensor was observed to be highly rigid and thermodynamically stable to become an interesting candidate that may form the basis for development of general nucleic acid detection platforms in the near future.

## Experimental Section

Experimental section and further analysis of in vitro synthesis and purification of pRNA 3WJ; monoconjugation strategy for AuNP–RNA 3WJ; photobleaching assay and analysis of the photobleaching traces; gold nanoparticle deposition on substrate; fabrication of the biosensor; EC‐SERS experimental setup; qRT‐PCR experiment and statistical analysis are provided in the Supporting Information.

## Conflict of Interest

P.G. is the licensor and consultant of Oxford Nanopore Technologies; cofounder of ExonanoRNA, LLC and its subsidiary Weina Biomedical LLC in Foshan; the cofounder of Shenzhen P&Z Bio‐medical Co. Ltd.

## Supporting information

Supporting Information
