# Supplementary material for: Single Functionalized pRNA/Gold Nanoparticle for Ultrasensitive MicroRNA Detection Using Electrochemical Surface‐Enhanced Raman Spectroscopy
Source: Adv Sci (Weinh). 2019 Dec 18;7(3):1902477. doi: 10.1002/advs.201902477 (PMC7001639; doi:10.1002/advs.201902477)
Supplement: Supplementary file 1 — Supporting Information [file ADVS-7-1902477-s001.pdf]

Copyright WILEY-VCH Verlag GmbH & Co. KGaA, 69469 Weinheim, Germany, 2019.

Supporting Information

for *Adv. Sci.*, DOI: 10.1002/advs.201902477

Single Functionalized pRNA/Gold Nanoparticle for Ultrasensitive MicroRNA Detection Using Electrochemical Surface-Enhanced Raman Spectroscopy

Taek Lee, Mohsen Mohammadniaei, Hui Zhang, Jinho Yoon, Hye Kyu Choi, Sijin Guo, Peixuan Guo, and Jeong-Woo Choi**

Copyright WILEY-VCH Verlag GmbH & Co. KGaA, 69469 Weinheim, Germany, 2019.

Supporting Information

Single Functionalized pRNA/Gold Nanoparticle for Ultra-Sensitive MicroRNA Detection Using Electrochemical Surface-Enhanced Raman Spectroscopy

Taek Lee [1,2,3,†], Mohsen Mohammadniaei [2,†], Hui Zhang [1], Jinho Yoon [2], Hye Kyu Choi[2], Sijin Guo[1], Peixuan Guo [1,] and Jeong-Woo Choi [2,*]*

Prof. T. Lee, Prof. H. Zhang, S. Guo, Prof. P. Guo

[1] College of Pharmacy; College of Medicine/Department of Physiology and Cell Biology/Dorothy M. Davis Heart and Lung Research Institute; Ohio State University, Columbus, OH 43210, USA
E-mail: guo.1091@osu.edu, Tel: +1-859-218-0128

Prof. T. Lee, Dr. M. Mohammadniaei, H. K. Choi, Dr. J. Yoon, Prof. J. -W. Choi

[2] Department of Chemical and Biomolecular Engineering, Sogang University, 35 Baekbeom-ro, Mapo-gu, Seoul 121-742, Republic of Korea
E-mail: jwchoi@sogang.ac.kr, Tel: +82-2-705-8480

Prof. T. Lee

[3] Department of Chemical Engineering, Kwangwoon University, 20 Kwangwoon-Ro, Nowon-Gu, Seoul 01897, Republic of Korea

[†] These authors contributed equally to this work.

S1. Material and reagents

The streptavidin-encapsulated Au nanoparticles (STV/AuNP, 3 nm) were purchased from Nanocs (USA). 6-MHA (6-mercaptohexanoic acid), RNase-free phosphate-buffered saline (PBS) (pH 7.4, 10 mM), (3-aminopropyl)triethoxysilane (APTES), 4-mercaptobenzoic acid (4-MBA), tris(2-carboxyethyl) phosphine hydrochloride (TCEP), Sephadex G100 resin, and serum albumin (BSA) were purchased from Sigma Aldrich (USA). ITO glass (2 × 1 cm; ~ 7.065 mm^2 active area) was purchased from G-mac (South Korea). The binding buffer (50 mM HEPES, 150 mM NaCl, 10 mM $MgCl_2 \cdot 6H_2O$) and the elution buffer (4 M Urea, 5 mM EDTA) were prepared for conjugation. The water used for all experiments was distilled and deionized using Millipore (Milli-Q) (DDW, >18 MΩ). DEPC-treated H_2O and all of the RNA oligonucleotides were synthesized and purified by the HPLC method and provided by Bioneer (South Korea), and the sequences are listed in Table S3.

S2. In vitro synthesis and purification of pRNA 3WJ

The pRNA 3WJa, 5′-end biotin-labeled pRNA 3WJb (Bio/3WJb), DNA 3WJb, 5′-end Cy3-labeled 3WJa (Cy3/3WJa), 5′-end methylene blue-labeled complementary miR155, 3′-end thiol-labeled 3WJc (MB/miR155-3WJc/SH), and miR-155 were chemically synthesized by Integrated DNA Technologies (IDT). A sephadex G100 binding RNA aptamer sequence was added to the 3′-end of the pRNA 3WJc (Fig. 2a). The dsDNA template for the sephadex RNA aptamer-pRNA 3WJc was prepared by annealing two reverse complementary ssDNA oligos chemicals synthesized by IDT. The sephadex RNA aptamer-pRNA 3WJc strand was synthesized by the in vitro transcription of the corresponding DNA template by T7 RNA polymerase.

S3. Mono-conjugation strategy for AuNP-RNA 3WJ

The RNA-aptamer-based conjugation technique was carried out for conjugating a streptavidin–gold nanoparticle (STV/AuNP) with a SEPapt/3WJ/Bio nanostructure. Fig. 1a describes the stepwise STV/AuNP-Bio/3WJ conjugation process, which was designed based on the Sephadex G100 recognized RNA aptamer. On the basis of our approach, we could expect the STV/AuNP-Bio/3WJb conjugation. In brief, the RNA-aptamer-based conjugation strategy has proved to control the amount of STV on each 3WJ RNA nanoparticle. This process was initiated by the aptamer recognition reaction between the surface of the Sephadex G100 resin and SEPapt/3WJ/Bio. The reaction mixture of SEPapt/3WJ/Bio (1 μM) and G100 resin (30 mg) were placed in the binding buffer and incubated for 12 hr at 4 °C with shaking. The SEPapt/3WJ/Bio immobilized G100 resins were then washed three times by centrifugation with the binding buffer. Subsequently, 300 μL of 1 μM streptavidin-coated gold nanoparticles (STV/AuNPs) were added to the SEPapt/3WJ/Bio on G100 resins, and reacted with gentle shaking at 4 °C in the dark for 6 hrs. The STV/AuNP was bound to Bio/SEPapt/3WJ on G100 resins through STV–biotin binding. Unreacted STV/AuNP was washed out three times by centrifugation with the binding buffer. Finally, the STV/AuNP-Bio/3WJb was released from the G100 resins by adding an elution buffer containing 4 M urea and 5 mM EDTA with gentle mixing for approximately 30 mins, and this step was repeated five times. The STV/AuNP-Bio/3WJb, SEPapt/3WJc, and DNA 3WJa fragments were dissociated in the elution buffer. To separate the STV/AuNP-Bio/3WJb and other 3WJ fragments from the G100 resin, the eluted samples were filtered with a spin column filter. Next, the STV/AuNP-Bio/3WJ b fragment was separated using a centrifugation filter (MWCO 10K, Amicon Ultra centrifugal filter). The specifically conjugated STV/AuNP-Bio/3WJb was then re-assembled with Cy3/3WJa and 3WJc for further experimentation.

S4. Photobleaching assay and analysis of the photobleaching traces

To perform the single-molecule photobleaching assay, the biotin-coated chamber was prepared by incubation of a flow cell using 1 mg/mL biotin-BSA (Sigma) for 15 min. The streptavidin-coated AuNP/Cy3-3WJ complexes were immobilized on the chamber surface through the streptavidin–biotin reaction with the concentration adjusted to produce discrete fluorescent spots. Unreacted complexes were washed out of the chamber before imaging by using TMS buffer (50 mM Tris-HCl, 10 mM $MgCl_2$, 100 mM NaCl). A 532 nm green laser was used for the excitation of Cy3. A 100× objective (NA 1.45, oil immersion) was used for single-fluorophore objective-type TIRF imaging, and a Cy3 filter cube (ET-Cy3, Chroma Technology Corp.) was used for Cy3 signal collection. The signals were recorded using an Andor iXon 887V electron-multiplied CCD camera. To prevent fast photobleaching, the flow cell was infused with an oxygen scavenger system (0.5% β-D-glucose, 10 mM β-mercaptoethanol, 0.2% glucose oxidase (Roche), and 0.25% catalase (Sigma)) during imaging. Sequential images were taken continuously with an exposure time of 300 ms. The recorded movie, which had more than 2000 frames, was analyzed by Andor IQ software (Andor Technology).

S5. Gold nanoparticle deposition on substrate

ITO-coated glass substrates were cleaned via sonication for 30 min using 1% Triton X-100 solution, DIW and ethanol sequentially, before treating in a basic piranha solution (1:1:5, H_2O_2: NH_3:H_2O) for 30 min at 80 °C. Finally, the substrates were rinsed by DIW and then dried under N_2 stream to obtain a clean ITO surface. Au nanopattern was electrochemically deposited on ITO substrates using a 0.5 mM $HAuCl_4$ aqueous solution containing 20 µl/ml Tween 20 as a structure-directing agent in 15 s. The reaction was conducted at a constant optimal potential of -1.25 V (vs. Ag/AgCl) while the deposition temperature was controlled to be maintained at 25 °C in an electric-heated thermostatic water bath. In order to remove any

surfactant traces that may have adsorbed on the developed pattern, the substrates were rinsed with DIW and then boiled with isopropyl alcohol for 5 min.

As shown in Fig. S1 (a – c) a well-istributed Au nanopattern on ITO glass was achieved in the -1.25 V while the density of Au nanostructures increased by increasing the potential along with the evidential agglomeration of particles. We also investigated the plasmonic properties of the AuNP/ITO electrodes prepared under the three different potentials of -1.25, -1.7 and -2 V (vs. Ag/AgCl) using 4-MBA as the Raman tag. Under the identical conditions, very low concentration of 1 pM 4-MBA dissolved in ethanol was immobilized onto the three electrode types via gold-thiol conjugation, in order to have an identical amount of 4-MBA on all of the three electrode types. After 2 hr of incubation at RT and rinsing with ethanol and DI water, electrodes were directed to the SERS measurement. As seen in Fig. S1 (e and f), those electrodes fabricated at -1.25 V represented the highest average SERS signal. However, the electrodes prepared at -1.7 and -2 V showed almost similar and lower SERS signals which might be due to the aggregation of NPs on the surface, following by weaker localized surface plasmon resonance (LSPR). As a result, AuNP/ITO electrodes fabricated under the constant potentials of -1.25 V were used for further sensing analysis.

S6. Fabrication of the biosensor

RNA degradation. 20 µl of 2 µM 5’-Thiol modified miR-155, which was also modified by Methylene Blue at its 3’ terminal, after being treated by TCEP to avoid double thiol bindings, was immobilized onto the GNP deposited ITO substrate. After incubation for 24 hr at 25°C in an 80% humid chamber, substrate was rinsed with PBS buffer (0.1 M NaCl, 10 mM phosphate, pH 7.4) and dried gently under the stream of nitrogen. Then 20 µl of the mixture of 3WJ-a and Bio/3WJ-b strands at 2 µM, which was diluted in the hybridization buffer (10 mM Tris-HCl, 100 mM KCl, and 1 mM $MgCl_2$, pH 7.4), was immobilized to the surface and incubated for 3 hr at 37◦C and 80% humidity. Then the surface was gently rinsed with DEPC

water and was backfield by immersing into the solution of 10mM 6-MHA for 2 hr at 37◦C in order to remove physically bound strands to the surface. After washing and drying process, 5 µl of the target RNA (miR-155 or others) diluted in the hybridization buffer was dropped onto the surface at different concentrations and incubated for 1 hr at 37◦C and 80% humidity. Then the sample was gently washed with DEPC water and dried by N_2 gas and either implemented for experiment or soaked into PBS buffer and kept at 4 ^{o}C in an appropriate dark humid chamber for further analysis. To detect the miR-155 using mono-conjugated RNA-AuNP, STV/AuNP-Bio/3WJb was reassembled with other functional strands such as MB/miR-155-3WJc/SH and 3WJa by an annealing process. Each piece of the strand played a specific role. 3WJa was used as a displacer for miR-155 detection through the strand displacement effect. STV/AuNP-Bio/3WJb was assisted in EC-SERS signal amplification. MB/miR-155-3WJc/SH was used for EC-SERS signal generation, as well as the immobilization and hybridization of miR-155, simultaneously.

S7. EC-SERS experimental set-up

All EC-SERS experiments were performed in the 500 µL PBS buffer solution at pH 7.4 using a potentiostat (CHI-660, CH Instruments, USA) controlled by general-purpose electrochemical system software and Raman NTEGRA spectra (NT-MDT, Russia) equipped with an inverted optical microscope and a liquid nitrogen cooled CCD detector. Raman spectra were recorded using an NIR-laser-emitting light at a wavelength of 785 nm, with an irradiation laser power of 3 mW on the sample plane and the laser spot size of 1 µm^2. A homemade three-electrode system consisting of AuNP/ITO as the working electrode, a platinum wire as the counter electrode, and Ag/AgCl as the reference electrode was set up. The EC-SERS cell chamber (Fig. S2) consisted of a distillation tube (10 mm in diameter, 20 mm in height) that was attached to the AuNP/ITO substrate by means of PDMS to simultaneously measure the Raman spectra and electrochemical behavior of the biosensor.

The Raman spectrum was extracted from 10 points from at least three different samples under the application of an electrode potential from −0.3 V to +0.3 V, and the mean result was used. A blank spectrum was acquired prior to each step, which allowed the absorbance to be subsequently measured.

S8. Quantitative real-time polymerase chain reaction (qRT-PCR) experiment

There primers of stem-loop RT primer, forward primer and universal reverse primer (Table S3) were designed and supplied by the Bioneer® (Korea). The primers were diluted in DEPC water and kept at -20 ^{o}C for the further experiments. Stem-loop RT primer was denatured by incubating at 65 ^{o}C for 2 min followed by incubation on ice. For the reverse transcription of miRNAs and generation of cDNA template, 1 µl of the denatured stem-loop RT primer was mixed with 1 µl of RNA template, 0.5 µl 10 mM dNTP mixture, 11.15 µl nuclease-free water, 4 µl 5X First-Strand buffer, 2 µl 0.1M DTT, 0.1 µl RNase OUT (40 U/µL) and 0.25 µl SuperScript III RT (200 U/µl). Subsequently, the total volume of the solution (20 µl) was loaded in the LightCycler® capillary and placed in a real-time PCR LightCycler instrument (Roche Diagnostics) following the thermocycler conditions: Pre-incubation for 30 min at 16 ^{o}C, then 60 cycles at 30 ^{o}C for 30 s, 42 ^{o}C for 30s and 50 ^{o}C for 1 s. Afterwards, the mixture was incubated at 85 ^{o}C for 5 min to inactivate the reverse transcriptase. For the cDNA amplification we used LightCycler® FastStart DNA Master^{plus} SYBER Green I and followed the manufacturer's protocol for the SYBR Green I master mix preparation. A mixture of 12 µl nuclease-free water, 4 µl SYBR Green I master mix, 1 µl of 10 µM miR-155 forward primer, 1 µl 10 µM miR-155 reverse primer was prepared and stored on ice. Then 2 µl of the RT product (synthetic cDNA) was added to solution to make it 20 µl. Next, the final solution was loaded into the capillary, sealed and placed in the thermocycler under the condition of: pre-incubation at 55 ^{o}C for 5 min followed by 95 ^{o}C for 5 min, then 40 cycles of amplification at 95 ^{o}C for 5 s and 65 ^{o}C for 10 s.

S9. Statistical analysis

The pre-processing of data such as normalization and baseline subtractions were performed using Origin software. Relative standard deviations and mean values were calculated based on the experimental data recorded from at least three identical samples unless otherwise mentioned in the figure captions. Statistical analysis was carried out using SPSS 16.0 software and the statistically significant data was considered for the probability (*P*) less than 0.05.

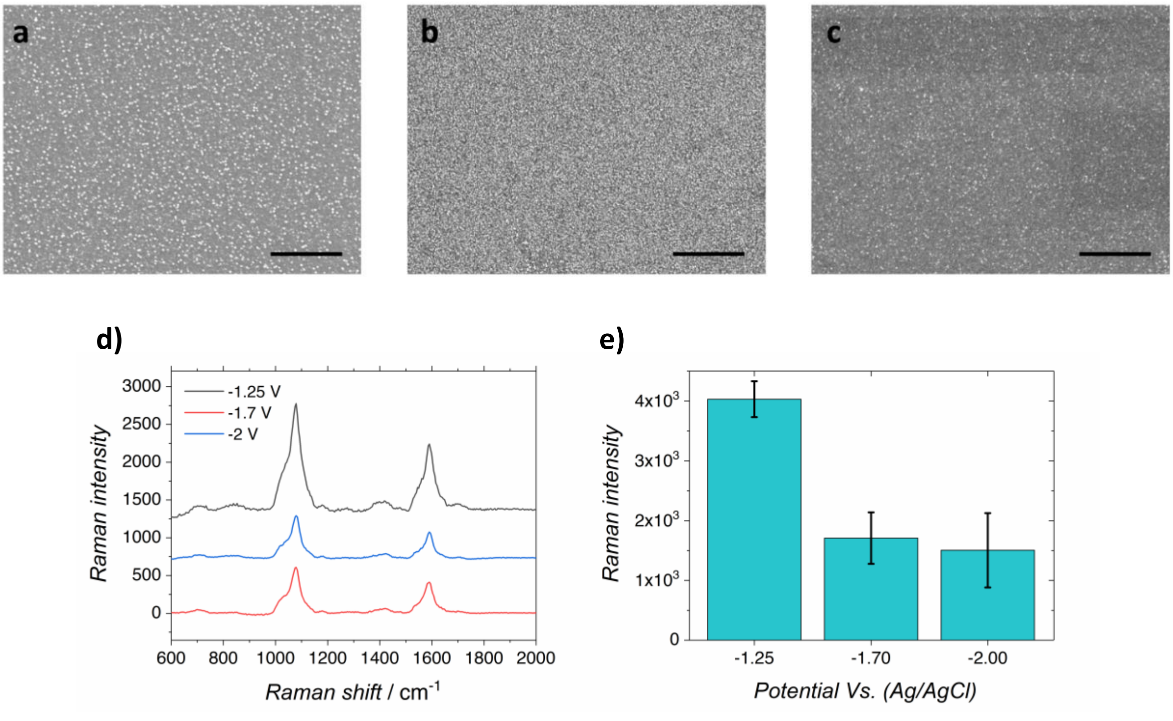

Fig. S1. SEM image of AuNP/ITO substrates fabricated at 0.5 mM $HAuCl_4$ aqueous solution containing 20 µl/ml Tween 20 for 15 sec in different potentials: (a) -1.25 V (b) -1.7 V and (c) -2 V. Scale bar is 1 µm. d) Typical SERS spectra of 4-MBA immobilized on fabricated AuNP/ITO electrodes at -1.25 V, -1.7 V and -2 V. e) Plotting the corresponding 4-MBA Raman intensity of the studied electrodes; SERS spectra were obtained using 785 nm laser and 1 sec exposure time and the data was analyzed based on the average signals recorded from 20 spots for three different samples.

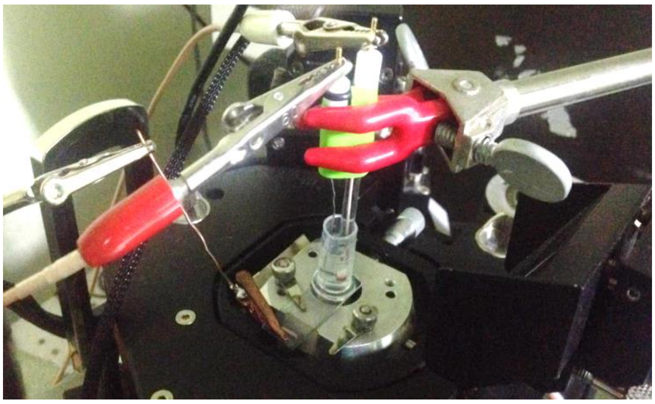

Fig. S2. Electrochemical surface enhanced Raman spectroscopy (EC-SERS) setup. The EC-SERS cell chamber consists of a distilled tube (8 mm in diameter × 20 mm in height) which is attached to the AuNP/ITO substrate by means of PDMS to simultaneously measure the Raman spectra and electrochemical behavior of the biosensor.

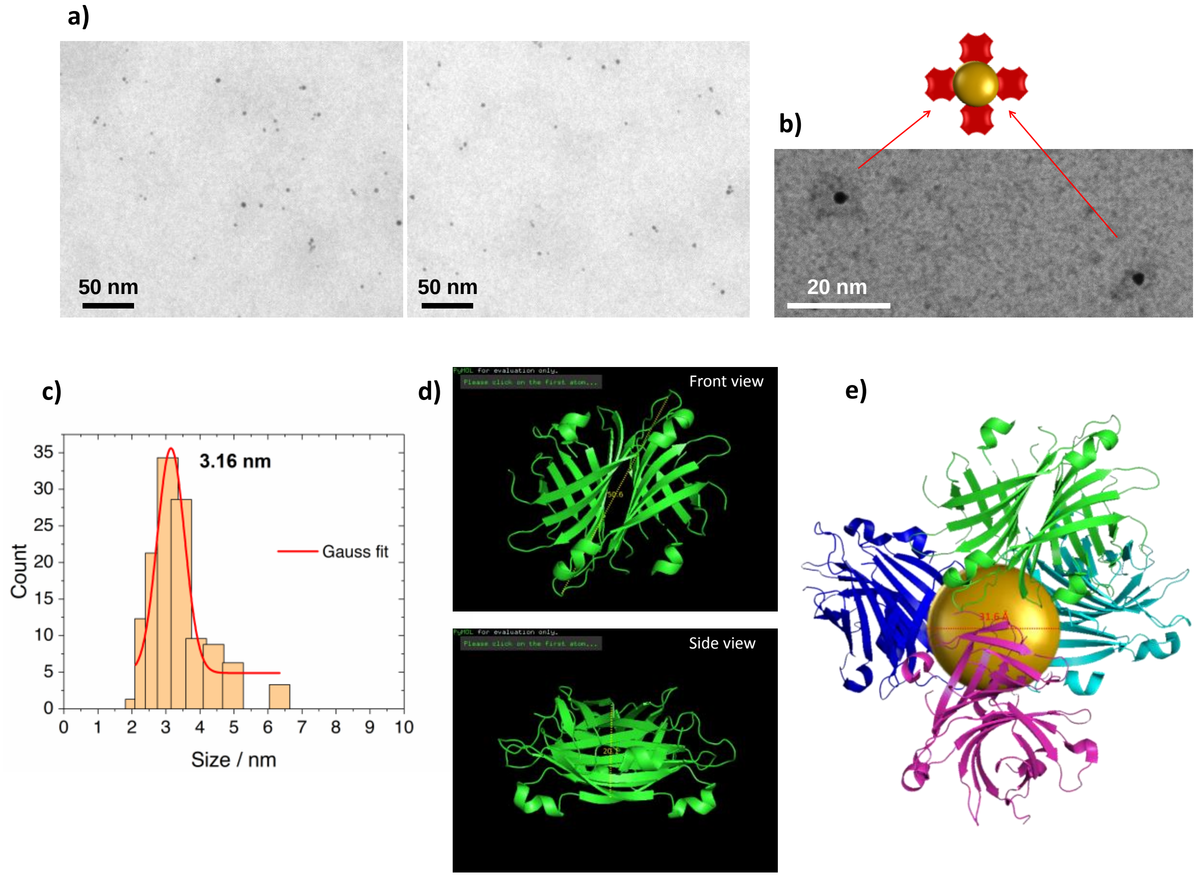

Fig. S3. a) Two representative TEM images from STV/AuNPs illustrating the mono-dispersed distribution of nanoparticles without aggregation. b) Magnified TEM image of STV/AuNP; the observed shadow around the AuNPs might represent the STV proteins. c) Size distribution plot of the STV/AuNPs; The statistical data was obtained using ImageJ software, however the actual size of STV/AuNP should be bigger due to the presence of STV on the surface. d) 3D view of a wild-type streptavidin protein (http://www.rcsb.org/structure/2G5L) demonstrating its dimension in angstrom;

images were obtained using PyMOL APBS software. e) Schematic diagram illustrating the spatial arrangement of STV on 3.16 nm AuNP; considering the size of STV and AuNP, maximum number of STV adsorbed on each AuNP is four.

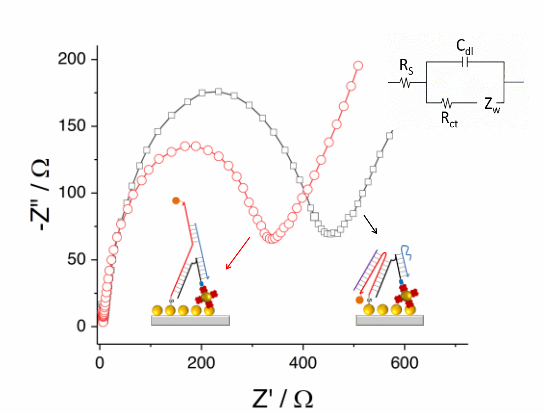

Fig. S4. Nyquist plots (Z′ vs. - Z″) plots recorded from 3WJ biosensor before (red; circle) and after (black; square) hybridization with 100 pM miR-155. The inset illustrates the equivalent Randles circuit model; The EIS experiment was conducted in PBS buffer (pH 7.4) containing 5 mM $[Fe(CN)_6]^{4-/3-}$ and 0.1 M KCl.

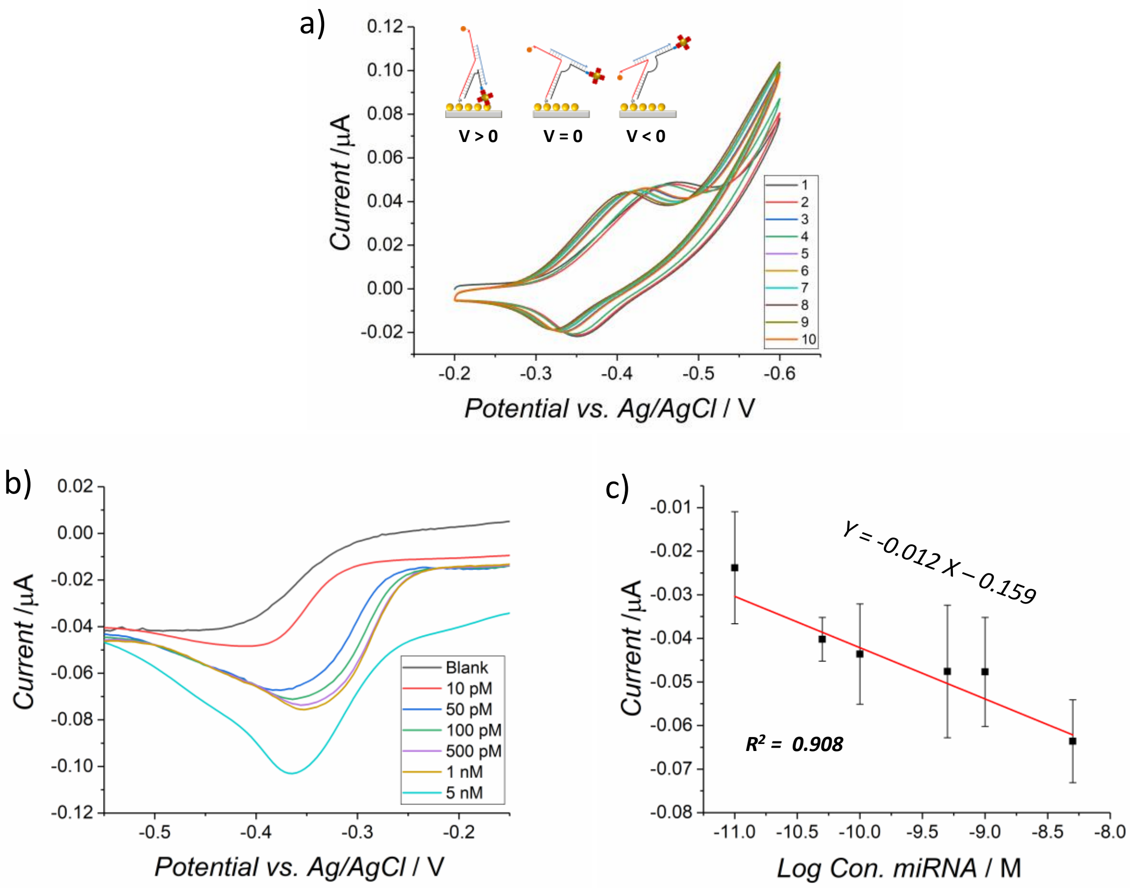

Fig. S5. a) Cyclic voltammograms recorded from 10 consecutive cycles of 3WJ biosensor after hybridization with 50 pM miR-155, inset represents the predictive schematic structures of orientation change of 3WJ biosensor under different potentials; b) DPV signals obtained from

3WJ biosensor after addition of different concentrations of miR-155; c) Corresponding calibration curve for oxidation current peak of MB as a function of logarithmic concentrations of miR-155. Electrochemical experiments were performed in PBS (pH 7.4). CV was recorded under the scan rate of 50 mV/s. Error bars were calculated based on three identical experiments.

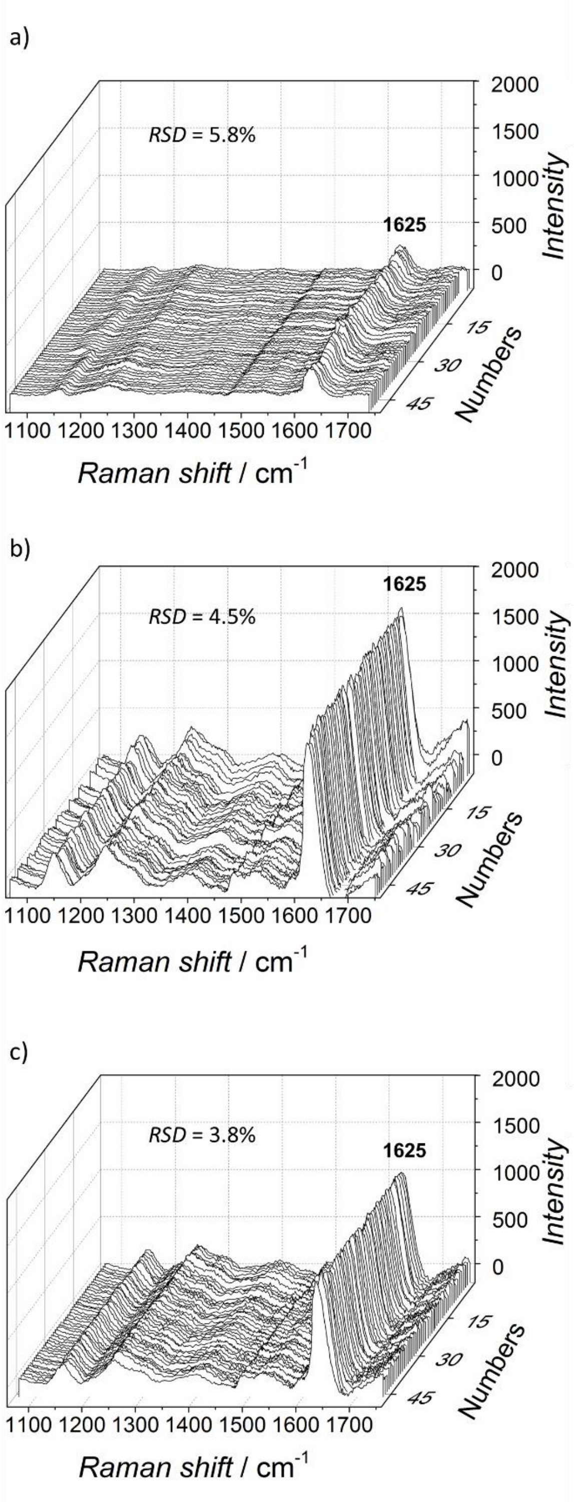

Fig. S6. EC-SERS spectra at +0.2 V extracted from 50 **hot spots** of the 3WJ biosensor before (a) and after being hybridized by b) 100 fM miR-155, c) 100 fM single-base mismatched miR-155.

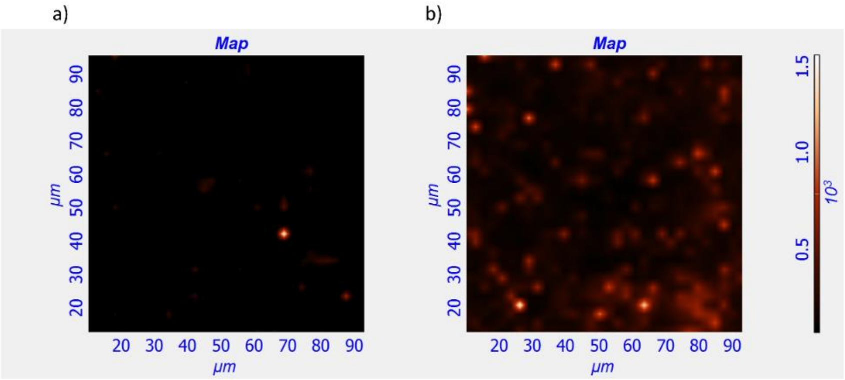

Fig. S7. EC-SERS map at +0.2 V showing hot spots of the 3WJ biosensor before (a) and after being hybridized by 10 pM miR-155 (b).

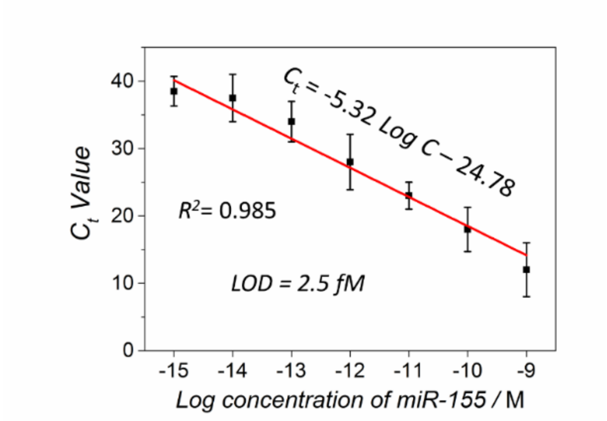

Fig. S8. Quantitative real-time PCR for the fluorescence monitoring of the amplification reaction triggered by different concentrations of miR-155 by plotting of the C_t value as a function of the miR-155 concentration. Data analysis was performed using LightCycler® Software 4.05 (Roche Applied Science).

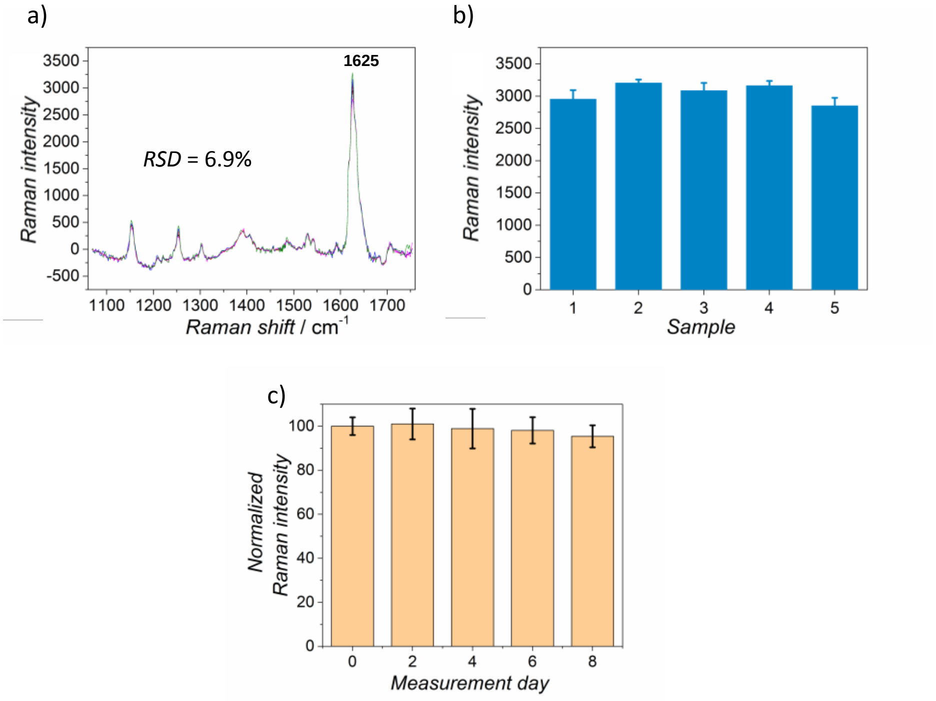

Fig. S9. a) EC-SERS spectra of five different 3WJ biosensors after detecting 100 pM miR-155 for the reproducibility analysis; b) EC-SERS signal intensities at 1625 cm^{-1} of five different samples averaged over 50 different spots; c) Stability test conducted from 5 sensors by recording and normalization of their EC-SERS signals every 2 days. The Raman signals were obtained and averaged from 50 random spots.

Table S1. Comparison study between different miRNA detection methods.

Detection method		Challenges	Detection steps	LOD	Detection time	Ref.
Other Techniques	DSN and AuNPs-based systems	Special enzymes needed, Unable to detect single mismatch	1	100 fM & 0.2 fM	~ 2 hr	[1]
	Cleavage-based methods	Labor intensive, Special enzymes needed	10	69.2 aM	> 2 hr	[2]
	qPCR / RT-PCR	Inefficiency in short primer hybridization, Difficult primer design, cross-hybridization	> 2	Femtomolar	~ 2 hr	[3]
	Isothermal signal amplification (QD-based)	require fluorescein-labeled recognition probes, circular templates, and complex procedure designs Multiple enzymes used, Cell cytotoxicity	5	0.1 aM	~ 3 hr	[4]
	Bio-barcode gel electrophoresis	Multistep process, Using highly toxic Potassium Cyanide	6	1 aM	~ 4 hr	[5]
Spectroscopy-based Biosensor (SERS)	Oblique angle vapor deposition (OAD) - prepared silver nanorod substrates	High (10s) laser exposure time, Low sensitivity, Low reproducibility due to the drop and dry process	1	µM level	~ 1 hr	[6]
	Unmodified silver nanorod array substrate	Non-specific bonding of none target strands, Low sensitivity	2	25 nM	< 1 hr	[7]
	Inverse Molecular Sentinel (iMS) Nanoprobes	Unable to detect single mismatch, Low reproducibility due to the particle agglomeration	1	~ 5 fM	~ 1 hr	[8]
	Plasmonic Nanowire Interstice Sensor	Cross contamination due to the necessity for a high accurate temperature control, Poor reproducibility and environmental instability resulting from the nanowire agglomeration	3	0.1 fM	~ 3 hr	[9]
	Cyclical nucleic acid strand-displacement polymerization (CNDP)	Multiple enzymes needed, Error-prone detection due to the multistep process	7	6.3 fM	~ 5 hr	[10]
Electrochemical-based Biosensors	Magnetobiosensor	Side reaction possibility, Probability of protein denaturation due to high temperature	4	0.4 fM	~ 3 hr	[11]
	3-mode electrochemical sensor	SWV signal is not as stable and reproducible as CV	2	5 aM	~ 2 hr	[12]
	Tandem polymerization and cleavage-mediated cascade system	Multiple enzymes, chemical reagents and probe modifications needed,	7	5 fM	~ 4 hr	[13]
	DNA tetrahedral scaffold	Difficult primer design, Complicated system, enzyme-based signal transduction	4	10 aM	~ 8 hr	[14]
	Carbon nanotubes and nanodiamonds	Difficult primer design and sophisticated labeling structure, Hybridization inefficiency between miRNA and labeling probe	2	1.95 fM	~ 2 hr	[15]
**EC-SERS**	3WJ biosensor	Needs improvement for multiple target detection	1	60 aM	1 hr	Present work

Table S2. Experimental data for the recovery of miR-155 in 5% and 10% human serum based on the measurement of EC-SERS signal intensity at 1625 cm^{-1}.

Sample	Concentration	Added (pM)	Found (pM)	Recovery (%)	RSD (%)
**Human serum 1**	5 %	1.0	1.06	106.00	6.9
**Human serum 2**	5 %	10.0	9.72	97.20	4.1
**Human serum 3**	5 %	100.0	99.86	99.86	5.7
**Human serum 4**	10 %	1.0	0.969	96.90	8.1
**Human serum 5**	10 %	10.0	10.41	104.10	7.2
**Human serum 6**	10 %	100.0	98.22	98.22	6.0

Table S3. Oligonucleotides sequences. Red character illustrates the mismatch location.

Oligo Name	Sequence (5' -> 3')
Aptamer template	GGA TCA ATC ATG GCA AAA AAG TCC GAG TAA TTT ACG TTT TGA TAC GGT TGC GGA ACT TGC
Aptamer forward primer	GTA TAA TAC GAC TCA CTA TAG GGC CGG ATC AAT CAT GGC AA
Aptamer reverse primer	GCA AGT TCC GCA ACC GTA TCA
miR-155	UUAAUGCUAAUCGUGAUAGGGG
Single mismatched miR-155	UUAAUGCUA**G**UCGUGAUAGGGG
miR-21	UAGCUUAUCAGACUGAUGUUGA
miR-141	UAACACUGUCUGGUAAAGAUGG
miR-143	UGAGAUGAAGCACUGUAGCUCA
SH/miR-155-3WJc/MB	Thiol/ GGAUCAAUCCCCUAUCACGAUUAGCAUUAA /Methylene Blue
3WJa	UCGUGAUAGGGGUGUAUGUGGG
Cy3/3WJa	Cy3/UCGUGAUAGGGGUGUAUGUGGG
Biotin/3WJb	Bio/CCCACAUACUUUGUUGAUCC
miR155-RT primer	GTTGGCTCTGGTGCAGGGTCCGAGGTATTCGCACCAGAGCCAACAC CCCT
miR155-forward primer	GTGGGTTAATGCTAATCGTGAT
miR155-reverse primer	GTGCAGGGTCCGAGGT

References

[1] B.-C. Yin, Y.-Q. Liu, B.-C. Ye, *J. Am. Chem. Soc.* **2012**, *134*, 5064; F. Degliangeli, P. Kshirsagar, V. Brunetti, P. P. Pompa, R. Fiammengo, *J. Am. Chem. Soc.* **2014**, *136*, 2264.

[2] Y. Zhao, L. Zhou, Z. Tang, *Nat. Commun.* **2013**, *4*, 1493.

[3] T. Nolan, R. E. Hands, S. A. Bustin, *Nat. Protoc.* **2006**, *1*, 1559; M. de Planell-Saguer, M. C. Rodicio, *Anal. Chim. Acta* **2011**, *699*, 134; S. Catuogno, C. L. Esposito, C. Quintavalle, L. Cerchia, G. Condorelli, V. De Franciscis, *Cancers* **2011**, *3*, 1877.

[4] Y. Zhang, C.-y. Zhang, *Anal. Chem.* **2012**, *84*, 224.

[5] H. Lee, J.-E. Park, J.-M. Nam, *Nat. Commun.* **2014**, *5*, 3367.

[6] J. D. Driskell, A. G. Seto, L. P. Jones, S. Jokela, R. A. Dluhy, Y. P. Zhao, R. A. Tripp, *Biosens. Bioelectron.* **2008**, *24*, 917.

[7] J. D. Driskell, R. A. Tripp, *Chem. Commun.* **2010**, *46*, 3298.

[8] H.-N. Wang, B. M. Crawford, A. M. Fales, M. L. Bowie, V. L. Seewaldt, T. Vo-Dinh, *J. Phy. Chem. C* **2016**, *120*, 21047.

[9] T. Kang, H. Kim, J. M. Lee, H. Lee, Y.-S. Choi, G. Kang, M.-K. Seo, B. H. Chung, Y. Jung, B. Kim, *Small* **2014**, *10*, 4200.

[10] H. Zhang, Y. Liu, J. Gao, J. Zhen, *Chem. Commun.* **2015**, *51*, 16836.

[11] S. Campuzano, R. M. Torrente-Rodríguez, E. López-Hernández, F. Conzuelo, R. Granados, J. M. Sánchez-Puelles, J. M. Pingarrón, *Angew. Chem. Int. Ed.* **2014**, *53*, 6168.

[12] M. Labib, N. Khan, S. M. Ghobadloo, J. Cheng, J. P. Pezacki, M. V. Berezovski, *J. Am. Chem. Soc.* **2013**, *135*, 3027.

[13] S. Liu, H. Gong, Y. Wang, L. Wang, *Biosens. Bioelectron.* **2016**, *77*, 818.

[14] Y. Wen, H. Pei, Y. Shen, J. Xi, M. Lin, N. Lu, X. Shen, J. Li, C. Fan, *Sci. Rep.* **2012**, *2*, 867.

[15] L. Liu, C. Song, Z. Zhang, J. Yang, L. Zhou, X. Zhang, G. Xie, *Biosens. Bioelectron.* **2015**, *70*, 351.
